# The use of abstract animations and a graphical body image for assessing pain outcomes among adults with sickle cell disease

**DOI:** 10.1016/j.jpain.2024.104720

**Published:** 2024-10-22

**Authors:** Julia A. O’Brien, Charles R. Jonassaint, Ektha Parchuri, Christina M. Lalama, Sherif M. Badawy, Megan E. Hamm, Jennifer N. Stinson, Chitra Lalloo, C. Patrick Carroll, Santosh L. Saraf, Victor R. Gordeuk, Robert M. Cronin, Nirmish Shah, Sophie M. Lanzkron, Darla Liles, Cassandra Trimnell, Lakiea Bailey, Raymona Lawrence, Leshana Saint Jean, Michael DeBaun, Laura M. De Castro, Tonya M. Palermo, Kaleab Z. Abebe

**Affiliations:** a Department of Acute and Tertiary Care, School of Nursing, University of Pittsburgh, Pittsburgh, PA, USA; b Department of Medicine, University of Pittsburgh, Pittsburgh, PA, USA; c Department of Pediatrics, Northwestern University Feinberg School of Medicine, Chicago, IL, USA; d Division of Hematology, Oncology and Stem Cell Transplant, Ann & Robert H. Lurie Children’s Hospital of Chicago, Chicago, IL, USA; e Lawrence S. Bloomberg, Faculty of Nursing, University of Toronto, Toronto, ON, Canada; f Child Health Evaluation Sciences, Research Institute, The Hospital for Sick Children, Toronto, ON, Canada; g Institute for Health Policy, Management & Evaluation, University of Toronto, Toronto, Canada; h Johns Hopkins Sickle Cell Center for Adults, Department of Psychiatry and Behavioral Sciences, Johns Hopkins School of Medicine, Baltimore, MD, USA; i Sickle Cell Center, Department of Medicine, University of Illinois at Chicago, Chicago, IL, USA; j Department of Internal Medicine, The Ohio State University, Columbus, OH, USA; k Sickle Cell Transition Program, Division of Hematology, Division of Pediatric Hematology/Oncology, Duke University, Durham, NC, USA; l Department of Internal Medicine, East Carolina University, Greenville, NC, USA; m Sickle Cell 101, San Jose, CA, USA; n Sickle Cell Community Consortium, Atlanta, GA, USA; o Jiann Ping Hsu College of Public Health, Georgia Southern University, Savannah, GA, USA; p Vanderbilt-Meharry Center of Excellence in Sickle Cell Disease, Vanderbilt University Medical Center, Nashville, TN, USA; q Department of Anesthesiology & Pain Medicine, University of Washington, and Seattle Children’s Research Institute, Seattle, WA, USA

**Keywords:** Sickle cell disease, Pain, Mental health, Ecological momentary assessment, eHealth, mHealth

## Abstract

Painimation, a novel digital pain assessment tool, allows patients to communicate their pain quality, intensity, and location using abstract animations (painimations) and a paintable body image. This study determined the construct validity of painimations and body image measures by testing correlations with validated pain outcomes in adults with sickle cell disease (SCD). Analyses used baseline data from a multisite randomized trial of 359 adults with SCD and chronic pain. Participants completed questionnaires on demographics, pain severity, frequency and interference, catastrophizing, opioid use, mood and quality of life, plus the Painimation app. Participants were categorized by selected painimations, and were split into groups based on the proportion of painted body image. Potential confounding was evaluated by age, gender, race, education, disability, site, depression, and anxiety. The ‘shooting’ painimation was strongly associated with daily pain intensity, pain interference, frequency, and severity. ‘Electrifying’ was associated with daily pain and opioid misuse, while greater body area in pain correlated with worse outcomes across all pain measures. Both painimations and body image measures correlated with validated pain outcomes, quality of life and mental health measures. This demonstrates animations and body image data can assess SCD pain severity, potentially with more accuracy than a 0–10 scale. Future research will explore whether Painimation can differentiate biological and psychosocial pain components.

**Perspective:**

This article presents the preliminary construct validity of Painimation in SCD by examining the associations of “painimations” and body area image data with daily e-diary and traditional self-report pain outcomes.

## Introduction

Pain is the primary reason for healthcare utilization in the US, with approximately 20.4% of adults experiencing chronic pain.^1^ Despite its prevalence,^2–5^ pain assessment in healthcare settings remains inadequate.^6^ This inadequacy is particularly evident in conditions like sickle cell disease (SCD), where pain is the primary reason for healthcare visits.^7^

Pain assessment is deceptively complex, as pain is a multidimensional experience encompassing sensory and emotional aspects that are difficult to communicate.^8^ However, in most clinical settings, pain is assessed using unidimensional measures, such as numeric rating or visual analog scales (NRS and VAS), that oversimplify the pain experience, reducing it to a single number.^6,9^ This oversimplification is especially problematic for SCD patients, who experience both nociceptive and neuropathic pain.^7,10^

SCD is a rare, inherited disorder characterized by vaso-occlusive events causing acute and chronic pain, often requiring hospitalization.^11,12^ Despite known contributors to chronic pain in SCD, many patients have intractable pain with no obvious cause, and there are limited methods for evaluating SCD pain beyond the NRS and VAS.^7,13,14^ This highlights the need for more comprehensive pain assessment tools, particularly for complex conditions like SCD.

Several multidimensional patient-reported outcome tools have been used in an attempt to understand patient-reported pain descriptors and how they relate to the biology of SCD pain. These include the PAINReportIt,^15^ that leverages the McGill Pain Questionnaire (MPQ),^16^ neuropathic pain assessments, such as the PainDETECT,^17^ and emerging PROMIS measures.^18,19^ Studies using these tools have found that SCD patients often select both nociceptive and neuropathic pain descriptors, suggesting mixed pain mechanisms. For instance, one study using PAINReportIt found SCD patients selected an average of 4.5 neuropathic descriptors and 6.8 nociceptive descriptors.^15^ Words like “shooting,” “burning,” and “tingling” are frequently associated with neuropathic pain, while “throbbing” and “cramping” are typically nociceptive descriptors.^20^ Notably, 25–40% of SCD patients scored positive for neuropathic pain on specific screening tools.^20,21^

While these tools provide valuable insights into pain biology in SCD, their clinical application remains limited. This may be due to respondent burden and inconsistent correlation with clinical outcomes.^22,23^ Further, these multidimensional measures are often complex and rely on language that alienate individuals with communication difficulties,^24,25^ leading to misinterpretation, discordance, and loss of trust.^26–28^ Thus, current approaches to pain assessment may compromise quality care, particularly for underserved populations.^9,29,30^

Assessing pain qualities is challenging. Adjectives are highly subjective and many patients incorporate imagery and metaphor to more fully capture their pain^31^ but metaphors are impossible to quantify. Previous studies show that patients value measures that use images to represent pain, however validation data is limited^32,33^ and all of the currently available measures use static images.^34–37^ We previously developed an electronic tool called Painimation, which allows patients to communicate the quality, intensity, and location of their pain using abstract animations and a paintable body image.^29,38^ Painimation may better assess SCD pain quality and type by capturing characteristics that static images, words, and numeric scales miss.^14^

The objective of the current study was to demonstrate preliminary construct validity of Painimation in SCD by examining the associations of painimations (i.e., pain animations) and body area image data with daily e-diary and traditional self-report pain outcomes in a large cohort of adults with SCD. Based on a previous study of patients with chronic pain, we hypothesized that the “electrifying” and “shooting” painimations would be associated with worse pain outcomes, particularly higher VAS scores and pain severity, compared to the other painimations, and the “throbbing” animation would be associated with better pain outcomes.^29^ In addition, we hypothesize that greater total body area in pain will be associated with worse pain-related outcomes.

## Methods

### Study design

The Cognitive Behavioral Therapy and Real-time Pain Management Intervention for Sickle Cell via Mobile Applications (CaRISMA) trial, a multicenter study conducted at seven comprehensive sickle cell centers, aimed to compare the effectiveness of two mobile phone-delivered programs in reducing pain symptoms in patients with SCD. The trial enrolled participants through seven academic medical centers and over 25 community-based organizations that focus on SCD. This study presents a secondary analysis of the blinded baseline data from the CaRISMA trial.^39^

### Ethics approval and consent to participate

All methods in this study were performed in accordance with the Declaration of Helsinki. Ethical approval was obtained from the Institutional Review Board (IRB) Committee of the University of Pittsburgh. Participation in the study was voluntary and written informed consent was obtained from the participants prior to enrolling in the study.

### Inclusion and exclusion criteria

Participants in the study included English-speaking adults aged 18 years or older with any SCD genotype who reported chronic pain (defined as experiencing pain at least four days a week for the past three months or longer) and/or were prescribed long-acting or daily opioid medication for pain. Ownership of a smartphone was a requirement for all participants. Those who did not meet the chronic pain criteria or chose not to participate in either intervention arms had the option to join a non-intervention, observational comparison arm, which is not included in the current study.

### Recruitment, enrollment, and randomization

The study aimed to recruit a sample size of 350 participants using a hybrid enrollment strategy that combined in-person and web-based methods. This approach allowed eligible patients from across the United States to enroll, allowing for a more representative sample of the national adult SCD population. In-person recruitment took place at seven clinical academic sites, while remote, web-based enrollment involved partnerships with the University of Pittsburgh and various community-based organizations (CBOs) affiliated with the Sickle Cell Community Consortium (SCCC), including Sickle Cell 101 (SC101), Sickle Cell Warriors (SCWarriors), Children’s Sickle Cell Foundation, and 25 other affiliated CBOs across the US.

### Study recruitment and enrollment

To identify SCD patients with chronic pain, electronic, tablet-administered screening kiosks were used at enrollment sites and in-person CBO events. Remote enrollment allowed patients to access the screening kiosk through a web link on any internet-enabled device. Clinical sites and CBOs promoted the study through various channels such as websites, social media groups, email listservs, and patient/family meetings. Interested individuals accessed the screening kiosk, watched an explanatory video and slideshow, and completed six consent comprehension questions. Those who successfully completed the questions and wished to participate electronically signed the consent form.

### Measures

#### Painimation^29,38^

**Fig. 1.**
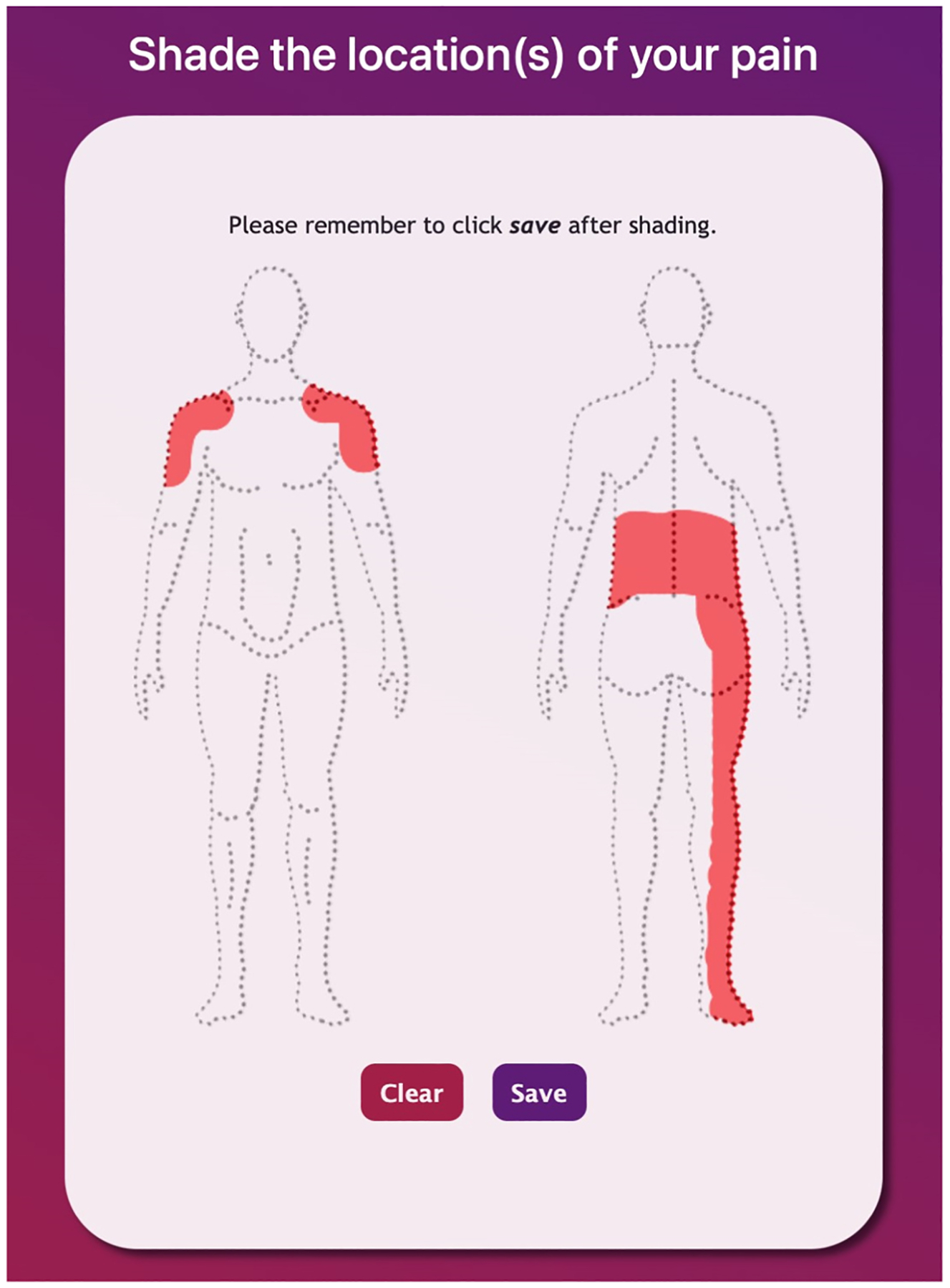
Example of the Painimation body map.

**Fig. 2.**
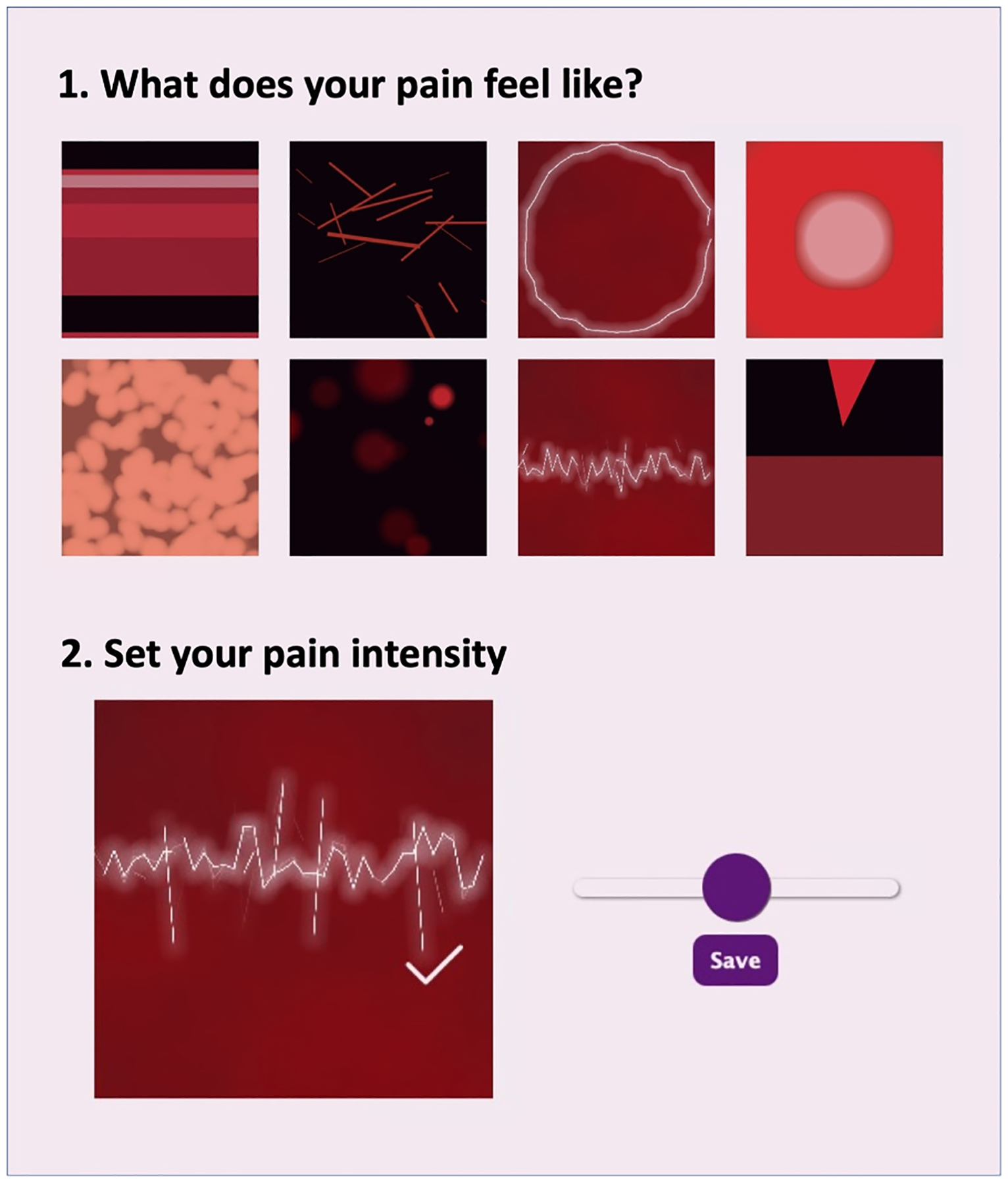
Examples of the Painimation pain-animations.

Painimation is an electronic pain assessment tool designed to measure pain location, quality, and severity using “pain animations,” or painimations. Patients use a paintable body image to indicate the areas affected by pain (Figure 1). After indicating pain location, patients can choose from eight painimations to indicate the specific pain qualities that best represent their pain experience. These painimations can be adjusted in size, speed, and saturation to accurately represent the intensity of pain using an adjustable sliding scale (see Figure 2) that controls the animation. The painimations are derived from the McGill Pain Questionnaire Short Form^16^ and were created using a human-centered design process that included patients with acute and chronic pain, clinicians, clinical researchers, and design students as key stakeholders in the process. The finalized painimations loosely refer to the following pain qualities; “pounding,” “shooting,” “throbbing,” “tingling,” “cramping,” “burning,” “stabbing,” and “electrifying.” These animations were selected based on the most commonly used descriptors chosen by patients and clinicians during the human-centered design process. More information about how Painimation was created can be found in previously published papers.^38,40^ An intensity score ranging from 0 to 100 is generated based how the painimation is adjusted (these data are not included in the current analyses). A maximum of three painimations could be selected. This was to limit the data to the most prominent pain qualities participants were experiencing and narrow the number of potential false associations with outcomes. The body area affected by pain assessment calculates the percentage of body area in pain based on the proportion of selected pixels.^29,38^

Outcomes for this study included average daily pain, the proportion of “happy” mood days, pain interference, ASCQ-Me pain frequency, ASCQ-Me pain severity, pain catastrophizing, and opioid misuse.

#### Electronic diary — Daily pain intensity and mood

**Fig. 3.**
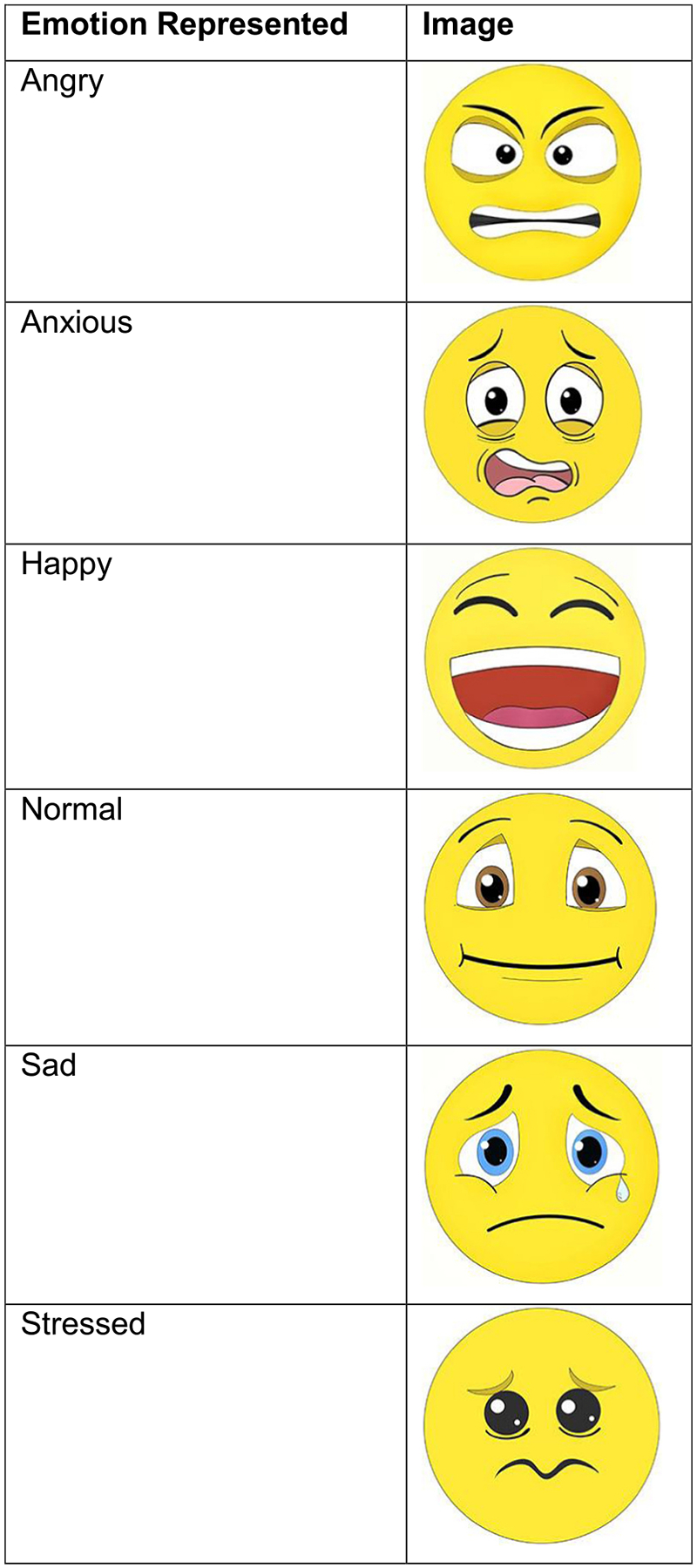
Daily mood data collection tool.

Participants used a mobile-adaptive web app to record their daily pain and mood. For pain intensity, they used a scale of 0 (no pain) to 10 (worst imaginable pain). To assess mood, we used an exploratory measure in which participants selected one of six emoji options representing angry, anxious, happy, normal, sad, or stressed states (Figure 3). These entries were completed once daily during a 2-week baseline period. For analysis purposes, we calculated the mean pain intensity over this baseline period. The mood outcome was the proportion of “happy” mood entries over a 2-week period.

#### Pain interference

PROMIS^®^ Pain Interference^19^ evaluates the impact of self-reported pain on various areas of an individual’s life, encompassing social, cognitive, emotional, physical, and recreational activities that may be hindered by pain. Participants rate how much pain has interfered with these aspects of their life over the past 7 days from 1 to 5 (with 1 representing “not at all” and 5 representing “very much”). Raw scores range from 8 to 40 but are transformed into T-Scores with a mean of 50, a standard deviation of 10, and a range of 0 to 100. Higher scores indicate more pain interference during everyday activities.

#### Adult Sickle Cell Quality of Life Measurement Information System (ASCQ-Me)^41^

This SCD-specific quality-of-life measure assesses various aspects of patients’ healthcare experience, emotional reactions to stress, and social relationships. In our study, we included ASCQ-Me measures of pain frequency, pain severity, emotional functioning and social impact. The ASCQ-Me subscales use Likert scales of varying ranges to measure the outcome of interest; the sum of each subscale is then transformed into a T-Score with a mean of 50, and a standard deviation of 10. Higher scores indicate better health outcomes related to pain, emotional functioning, and social impact.

#### Pain Catastrophizing Scale (PCS)^42^

This is a self-report measure used to assess catastrophic thinking related to pain. The PCS contained 13-items that use a 5-point Likert scale ranging from 0 – 4, representing “not at all” to “all the time” in response to items that measure the degree to which catastrophic thinking occurs during pain experiences. Total scores range from −0 – 52, with higher scores indicating a greater degree of catastrophizing.

#### Current Opioid Misuse Measure (COMM)^43^

This scale is a self-report tool used to track signs and risk factors of current aberrant drug-related behaviors in chronic pain patients undergoing opioid therapy. It consists of 17 items rated on a five-point Likert scale, with scores ranging from 0 to 68. Higher scores indicate a higher risk of opioid misuse.

Along with demographic variables, symptoms of depression and anxiety were measured and included as covariates in multivariate models.

#### Patient Health Questionnaire^44^

Depressive symptoms were measured using a screening protocol derived from the 9-item depression scale from the Patient Health Questionnaire, which is a scale of symptoms of major depressive disorder. Each item of the PHQ is rated on a scale from “0” (not at all) to “3” (nearly every day). All participants answered the first two items (PHQ-2), with further questions asked if scored above zero. Participants recruited from non-clinical settings also were not asked the 9th item (regarding suicidal ideation). The total scores PHQ-8 scores range from 0 to 24, and on the on the PHQ-9 range from 0 to 27 with cut-off points indicating mild (5), moderate (10), moderately severe (15), and severe (20) depression.^45^ For participants who affirmed suicidal ideation on the PHQ-9, a suicide risk management protocol was initiated. Further assessment of duration and acuteness of self-harm ideation, and severity of suicide risk was completed, based on the risk level the participant or 911 was called, and the local site suicide prevention plan was executed.

#### Generalized Anxiety Disorder Scale^46^

The measure assesses the severity of anxiety symptoms using a total score derived from seven items. Scores on the GAD-7 range from 0 to 21, with cut-off points of 5, 10, and 15 indicating mild, moderate, and severe anxiety levels, respectively.^47^ Participants in the parent study who affirmed no symptoms of anxiety on the GAD-2 did not complete the full GAD-7.

### Analysis

Descriptive statistics were calculated to summarize key characteristics and features of the data. The following descriptive statistics were computed for the variables of interest: mean, standard deviation (SD), median, minimum, and maximum values. Additionally, frequencies and percentages were calculated for categorical variables. Continuous variables were examined for normality using visual inspections of histograms and normal probability plots. Skewness and kurtosis values were also assessed to determine the distributional characteristics of the variables. For categorical variables, frequency distributions were examined to identify the number of observations falling into each category.

We tested the construct validity of Painimation by testing the association between different Painimation characteristics and pain and quality of life outcomes. Specifically, we examined the associations between each Painimation selection, and total body area image (TBI) group with the following pain and quality of life outcomes: mean daily pain intensity, proportion of days happy, pain interference, pain frequency, pain severity, pain catastrophizing, and opioid misuse. Regarding total body area, we split the participants into TBI groups based on the median: “< 9.8%” and “> 9.8%” in order to assess group differences in other pain and psychosocial outcomes.

Regression modeling was done in a stepwise fashion: first, univariate linear regression models were used to assess the relationship between each group (defined by how they entered their pain quality and body surface area affected on Painimation) and each outcome of interest. Second, for multivariable models we tested all potential covariates for their association with the exposure group. The potential covariates were: baseline age, gender, race/ethnicity, education, participant type (clinic vs. virtual), and disability status (self-report receipt of disability benefits. Due to the strong influence of depression/anxiety on pain outcomes in SCD,^48^ we examined baseline PHQ-9, PHQ-2, GAD-7, and GAD-2 scores as potential confounders. Both continuous and dichotomized baseline scores (e.g. GAD-2 > 1) were considered. Continuous GAD-7 scores were not significantly associated with any Painimation, thus, only dichotomous GAD-2 scores were included in the final models. Univariate models were assessed at the.05 level. The multivariable models were then run with only the covariates that were associated with the exposure group at p < .05. All statistical tests were conducted with a 5% type I error rate. To correct for multiple comparisons, a Bonferroni correction of .0036 was applied to the univariate analyses. The multivariate analyses are exploratory at this stage, and we did not apply a correction.

## Results

We enrolled 359 adults, mean age 36.3 (SD = 10.5), 66% female, 93% Black race, 36% were employed, and 75% had at least some college education. Further information about the demographics and baseline characteristics of the sample can be viewed in Table 1. During the two-week reminder period, patients completed an average of 8.7 pain diary entries (SD = 4.1), and the average pain intensity was 4.4 (SD = 2.5) using a 0 – 10 scale. In our sample, the mean ASCQ-Me: Pain Episode Severity and Frequency T-scores were 47.3 (SD = 13.3) and 48.3 (SD =12.3), respectively, indicating that this sample had similar pain episode severity and frequency relative to the general SCD population. Examining mental well-being, 39.5% of the sample had high symptoms of depression (PHQ-9 ≥ 10), and 70% of the sample had baseline GAD-7 scores > 0. T-tests and non-parametric tests are available in Table 2, and Table 3 contains the t-tests and non-parametric tests for the total body area affected by pain. These results as well as the univariate and multivariate models are summarized in the following section.

### Pounding

In univariate models, the “pounding” painimation was associated with pain current opioid misuse (mean difference = 2.733; p < 0.001). This difference was still significant at a.05 level (Mean difference = 1.54, p = 0.043) after adjusting for the PHQ and GAD2 > 0 covariates, which were significantly associated with the “pounding” painimation.

### Shooting pain

In univariate models, the “shooting” painimation was strongly associated with all outcomes (all p < .001) except for the proportion of “happy” mood days and opioid misuse (p = .004). Among the other variables of interest, only clinic site, PHQ-9 and GAD-7 were associated with shooting pain and were included in subsequent multivariable analyses. In multivariable models controlling for site, PHQ-9 and GAD-2 > 0, there were differences for average daily pain (Mean difference = 0.82; p = 0.010), pain interference (mean difference = 1.46; p = 0.033), ASCQ-Me pain frequency (Mean difference = 3.03; p = 0.030), and ASCQ-Me pain severity (Mean difference = 3.311; p = 0.024).

### Cramping

In univariate models, the “cramping” painimation was strongly associated with pain interference (mean difference = 2.5972; p < 0.001) and ASCQ-Me pain severity (mean difference = 4.707; p < 0.001). There were no strongly associated covariates that needed to be controlled for in multivariate models.

### Throbbing

In the univariate models, there were no significant associations between the “throbbing” painimation and pain outcomes after correction for multiple comparisons.

### Tingling

In univariate models, the “tingling” painimation had no significant associations with any pain outcomes after correction for multiple comparisons.

### Burning

In univariate models, the “burning” painimation had no significant associations with any pain outcomes after correction for multiple comparisons.

### Electrifying

In univariate models, the “electrifying” painimation was significantly associated with average daily pain (mean difference = 1.236, p < .001) and current opioid misuse (mean difference = 2.329, p < .001). The PHQ-2, self-efficacy, gender, education, employment, race, and GAD-2 were all significant covariates; after adjusting for these variables the only difference remaining was average daily pain (Mean difference = 0.980, p = 0.011).

### Stabbing

In univariate models, the “stabbing” painimation was significantly associated with ASCQ-Me pain frequency (mean difference = 4.286, p = .002). Disability status was a significant covariate; after adjusting for disability, significant findings remained for ASCQ-Me pain frequency (3.6526; p = 0.009).

### Total body area affected by pain

Greater body area scores were associated with worse outcomes on all measures in univariate analyses (all p < 0.05; Table 3. After controlling for age, PHQ, and participant type (clinic vs. virtual), greater body area score was associated with increased daily pain intensity (mean difference=1.18; p < 0.001), pain interference (mean difference=2.87; p < 0.001), ASCQ-Me pain frequency (mean difference=3.91; p≤0.005), and ASCQ-Me pain severity (mean difference=4.50; p = 0.002).

## Discussion

Individuals with SCD often struggle to convey their pain to healthcare providers, necessitating enhanced methods for accurate pain communication and treatment.^49–52^ The current tools often fall short in assessing patient-reported symptoms, compromising quality care.^53^ To address this, we introduced Painimation, a pain communication approach using abstract animations, or “painimations”.^38^ Previous studies show that painimations correlate with pain diagnosis and traditional pain scales, indicating their potential diagnostic value and efficacy.^29^ To our knowledge, this is now the largest study to test the use of an animation-based pain assessment tool in a chronic pain population.

Consistent with prior research in other pain populations,^29,54–56^ current data shows that pain quality is associated with the severity of pain symptoms in SCD. The study provides initial evidence of the construct validity of Painimation among adults with SCD, showing cross-sectional associations between the type of painimations chosen and measures of pain severity, disability, and opioid misuse. Overall, the “shooting” painimation was the most significantly associated with pain-related outcomes and was associated with depression and anxiety symptoms. If these data are replicated, this may be evidence to suggest the most physically and emotionally severe type of pain in SCD is characterized by the “shooting” painimation. In another study examining the association between pain quality and other pain characteristics among older adults, “shooting” pain was also one of the sensory-related qualities most associated with moderate to severe pain.^57^ “Shooting” is generally used as a descriptor of neuropathic pain^15,58^ but is also associated with nociceptive pain.^59^ Although the etiology of “shooting” pain and its treatment implications remain unclear, current and existing data suggest that “shooting” pain in SCD represents a more severe and difficult-to-treat pain phenotype compared to descriptors like “throbbing,” which may be solely associated with nociceptive pain.

Indeed, the “throbbing” painimation was endorsed by over 40% of participants and was one of the three most often selected. However, there were no robust associations between “throbbing” and pain severity or other psychological outcomes. What may be captured by this “throbbing” painimation is a low level, every day, chronic SCD pain, that is experienced in combination with other pain qualities or painimations.

For instance, other painimations, “electrifying” and “cramping” were associated with more severe daily pain and pain interference, respectively. In addition, the “electrifying” and “pounding” painimations were the only qualities significantly associated with opioid misuse. Previous research has suggested that the sensory experience of pain is weakly associated with risk of opioid misuse.^60^ However, this research examined whether increased severity of sensory experiences was associated with risk of opioid misuse, rather than whether particular pain qualities increased opioid misuse.

From the current data, it does appear among the several painimations where we found associations, the “electrifying” painimation may be a pain descriptor of particular interest in this population. To our knowledge, no studies have specifically looked at “electrifying” pain in SCD before. In other patient populations, “electrifying” is more clearly associated with neuropathic pain,^61,62^ and in a previous study of Painimation in a general chronic pain population, the “electrifying” painimation was associated with PainDetect, a measure of neuropathic pain, as well as self-reported nerve damage.^29^ In the current study, a measure of neuropathic pain was not included. However, future research may find that some combination of “shooting” and “electrifying” may be associated with a neuropathic pain component that is equally as severe and perhaps more difficult to treat with opioids than nociceptive pain in this population.

### Association between pain location, quality, and health outcomes

This study confirmed prior research showing that the body area covered by pain is positively correlated with pain severity.^63,64^ While pixel-based pain assessment methods have been available for some time,^65^ there are few studies to date that examine the total body area in pain and how this relates to physical and psychosocial health outcomes. For example, similar to the current study, one study in fibromyalgia patients found using a pixel-based paintable body map that higher body data and its association with pain catastrophizing, and also found that higher total pain area was associated with increased pain catastrophizing.^66^ To our knowledge, only one other study has examined the use of digital body map in SCD, and this application utilized selectable regions and not a paintable pixel based approach.^67^ The prior study of N = 99 adults with SCD did not show a robust association between body surface area selected and pain intensity or pain quality,^67^ conflicting with the current study’s findings. Pixel-based approaches may capture smaller between patient differences. Further, the current study’s larger patient sample may have allowed for the detection of more associations across outcomes compared to the prior study.

### The connection between pain quality and mood

In the current study, controlling for depression and anxiety appeared to attenuate the association between the Painimation-determined pain quality and measures of pain interference and other pain-related outcomes. The current study also showed a connection between pain quality and pain catastrophizing. These data only further confirmed the well-known influence mood, affect, and other psychological factors have on the pain experience.^68^ Previous studies have demonstrated the connection between pain quality and depression and anxiety symptoms,^69,70^ pain catastrophizing,^71^ as well as type of pain (neuropathic vs. nociceptive)^58^ in other non-SCD populations. Few studies have examined the role of patient reported pain qualities in SCD. A literature review found a wide variety across studies in the language used to describe SCD pain quality.^72^ Connections between pain quality and chronic pain have been explored, but other areas understudied.^73^ A study of adults with SCD in the outpatient setting using the MPQ found patients reported pain descriptors consistent with both neuropathic and nociceptive, as well as descriptions of a continuous or chronic pain pattern.^15^ Other studies have used a pain-rating index score calculated from the 20 groups of verbal MPQ descriptors, but do not provide data on the specific characteristics of pain that are related to pain outcomes.^74,75^ As such, it is difficult from the existing literature to understand the specific characteristics of pain that are related to pain and mental health outcomes in SCD.

### Clinically significant changes in pain intensity

Assessing patients’ acute pain medication needs and treatment satisfaction is challenging for providers due to unreliable data, variations in pain assessment abilities, and the complexity of identifying pain changes.^76,77^ In the present research, a 1.05 difference was observed in the two-week numeric rating scale (NRS) daily diary between the groups experiencing “shooting” pain and those with “non-shooting” pain. This suggests that variations in reported pain quality align with the clinically significant threshold for pain changes previously documented.^78–80^ A pediatric pain investigation revealed that a decrease of 0.97 cm on the VAS and 0.9 on the NRS marked the minimum clinically significant improvement in pain. Pain scores surpassing 7.45 cm on the VAS or 7.5 on the NRS signaled a patient’s desire for pain medication.^78^ However, this benchmark isn’t consistently applied in clinical settings. One of the key challenges in pain assessment is the lack of consensus on what signifies a clinically meaningful reduction in pain using the 0–10 NRS or the VAS. Future studies might determine that providers find it simpler to recognize clinical progress when patients report a change in pain quality than a numeric change. It might be that recognizing shifts in pain quality and characteristics, instead of or alongside numeric changes, offers a more effective approach to determine patients’ needs for pain management and their treatment satisfaction.

### Limitations

Although our study provides valuable insights into the associations among pain quality, location, and pain-related outcomes in adults with SCD, there are some limitations to consider. These include a lack of objective outcome measures and reliance on self-reported data for depression and outcomes. Incomplete e-diary data may impact the validity of our findings, as only 299 of 359 had sufficient diary entries (3 +) to be included in the analysis. The cross-sectional design limits causal inferences. Additionally, we did not include measures of neuropathic pain, despite their importance in SCD.^38,40,81^ Finally, potential confounding may have occurred due to participants selecting multiple painimations without prioritization. These factors may impact the validity and generalizability of our findings.

## Conclusion

This study validates a nonverbal pain expression method for SCD patients, potentially addressing communication and literacy barriers in pain assessment.^82–84^ Future research should explore painimations’ associations with neuropathic pain, depression, racial stigma, and long-term outcomes in SCD. The technique may also benefit pediatric pain assessment. Ultimately, an assessment approach that does not rely on language or cultural understanding, and can be used across age groups, would improve pain management for patients with SCD and other chronic pain populations.

## Figures and Tables

**Table 1:** Demographics and Baseline Characteristics.

Characteristic		Total (N = 359)
**Enrolled via:**	Clinic	265 (73.8%)
	Virtual	94 (26.2%)
**Age at baseline (years)**	Mean (s.d.)	36 (10)
**Gender**	Male	119 (33.1%)
	Female	238 (66.3%)
	Prefer not to answer	2 (0.6%)
**Ethnicity**	Hispanic/Latino	15 (4.2%)
	Not Hispanic/Latino	308 (85.8%)
	Unknown	36 (10.0%)
**Race**	Black/African American	332 (92.5%)
	White	2 (0.6%)
	American Indian/Alaska Native	1 (0.3%)
	Multiple/other	15 (4.2%)
	Prefer not to answer	9 (2.5%)
**Education**	Some high school	22 (6.1%)
	Completed high school or equivalent	69 (19.2%)
	Some college	160 (44.6%)
	Completed college	61 (17.0%)
	Graduate studies	47 (13.1%)
**Employment status**	Yes, employed	132 (36.8%)
	No, not employed	63 (17.5%)
	Disability	164 (45.7%)
**Pain Interference (PROMIS—8a)**	Mean (s.d.)	62.6 (7.1)
**Proportion of Days in Happy Mood (2-week)**	Mean (s.d.)	10% (20)
**Average Daily Pain Intensity (2-week)**	Mean (s.d.)	4.4 (2.5)
**Painimation (% body area shaded)**	Mean % (s.d.)	13.5% (15.0)
**Suicidal Ideation (Patient Health Questionnaire (PHQ) item #9)**	No	175 (85.4%)
	Yes	30 (14.6%)
	Did not complete	154
**PHQ Level of Depression Severity**	Minimal (0–4)	35 (12.0%)
	Mild (5–9)	116 (39.7%)
	Moderate (10–14)	76 (26.0%)
	Moderately severe (15–19)	50 (17.1%)
	Severe (20–27)	15 (5.1%)
	Did not complete	67
**Generalized Anxiety Disorder (GAD—7) Severity**	Minimal (0–4)	46 (18.3%)
	Mild (5–9)	113 (45.0%)
	Moderate (10–14)	62 (24.7%)
	Severe (15–21)	30 (12.0%)
	Did not complete	108
**Adult Sickle Cell Quality of Life**	Mean (s.d.)	48.3 (12.3)
**Measurement System (ASCQ-Me) – Pain Episode Frequency**		
**Adult Sickle Cell Quality of Life**	Mean (s.d.)	47.3 (13.3)
**Measurement System (ASCQ-Me) – Pain Episode Severity**		
**Pain Catastrophizing Scale (PCS) Score**	Mean (s.d.)	9 (4)
**Current Opioid Misuse Measure**	Mead (s.d.)	9 (5)

**Table 2:** Univariates associations between Painimation and pain outcomes.

	Painimations Selected															
	Pounding		Shooting		Cramping		Throbbing		Tingling		Burning		Electrifying		Stabbing	
Variables (M, SD)	No N = 307 85.5%	Yes N = 52 14.5%	No N = 213 59.3%	Yes N = 146 40.7%	No N = 209 58.2%	Yes N = 150 41.8%	No N = 212 59.1%	Yes N = 147 40.9%	No N = 321 89.4%	Yes N = 38 10.6%	No N = 266 74.1%	Yes N = 93 25.9%	No N = 268 74.7%	Yes N = 91 25.3%	No N = 251 69.9%	Yes N = 108 30.1%
Avg 0–10 pain, daily diary	4.30 (2.52)	4.80 (2.49) p= .211	**3.94** **(2.46)**	**4.99** **(2.48)** **p < .001**	4.06 (2.61)	4.76 (2.36) p = .017	4.12 (2.49)	4.74 (2.52) p = .037	4.36 (2.55)	4.50 (2.24) p = 0.740	4.22 (2.54)	4.80 (2.43) p = .0703	**4.06** **(2.52)**	**5.29** **(2.30)** **p < .001**	4.14 (2.56)	4.94 (2.33) p = .010
Proportion of “happy” days, daily diary	0.12 (0.18)	0.09 (0.14) p = .263	0.11 (0.16)	0.12 (0.20) p = .444	0.12 (0.18)	0.10 (0.18) p = .46	0.11 (0.17)	0.12 (0.19) p = .455	0.11 (0.17)	0.11 (0.20) p = 0.936	0.12 (0.19)	0.10 (0.14) p = .307	0.12 (0.17)	0.10 (0.19) p = .468	0.12 (0.17)	0.10 (0.19) p = .613
PROMIS Pain Interference	**62.1** **(7.25)**	**65.2** **(5.63)** **p < .001**	**61.2** **(7.79)**	**64.5** **(5.46)** **p < .001**	**61.5** **(7.24)**	**64.1** **(6.67)** **p < .001**	62.2 (7.33)	63.2 (6.77) p = 0.184	62.3 (7.22)	64.7 (5.81) p = .022	62.1 (7.43)	63.9 (5.96) p = .017	**62.0** **(7.31)**	**64.4** **(6.19)** **p = .003**	62.1 (7.29)	63.7 (6.57) p = .033
ASCQ-Me Pain Frequency	48.1 (12.6)	49.5 (10.9) p = .382	**46.0** **(13.2)**	**51.5** **(10.1)** **p < .001**	47.0 (13.0)	50.1 (11.1) p = .014	48.0 (12.8)	48.7 (11.7) p = 0.581	48.0 (12.4)	50.8 (12.1) p = 0.176	47.3 (12.4)	51.1 (11.7) p = .010	47.3 (12.7)	51.2 (10.6) p = .004	**47.0** **(12.7)**	**51.3** **(10.9)** **p = .001**
ASCQ-Me Pain Severity	46.8 (13.5)	50.1 (11.8) p = .073	**44.7** **(14.4)**	**51.1** **(10.4)** **p < .001**	**45.3** **(14.0)**	**50.0** **(11.8)** **p < .001**	46.4 (13.9)	48.5 (12.4) p = 0.138	47.1 (13.5)	49.2 (11.9) p = 0.299	46.5 (13.7)	49.4 (12.1) p = 0.056	**46.2** **(13.8)**	**50.5** **(11.2)** **p = .003**	46.1 (13.9)	50.0 (11.6) p = .006
Pain Catastrophizing Scale	9.07 (4.23)	10.5 (3.18) p = .006	**8.69** **(4.20)**	**10.1** **(3.86)** **p < .001**	8.99 (4.23)	9.68 (3.95) p = .112	9.23 (4.12)	9.34 (4.15) p = 0.806	9.23 (4.22)	9.68 (3.24) p = 0.431	8.99 (4.17)	10.1 (3.88) p = .021	9.03 (4.12)	9.99 (4.08) p = .056	9.19 (4.08)	9.47 (4.25) p = .561
COMM Opioid Misuse	**8.46** **(5.23)**	**11.2** **(5.95)** **p = .003**	8.18 (5.35)	9.84 (5.37) p = .004	8.30 (5.51)	9.63 (5.20) p = .021	8.84 (5.69)	8.88 (5.01) p = 0.947	8.86 (5.50)	8.79 (4.70) p = 0.929	8.62 (5.50)	9.52 (5.14) p = .159	**8.26** **(5.19)**	**10.6** **(5.72)** **p < .001**	8.70 (5.40)	9.21 (5.46) p = .415

Note: Results that are significant at a.0036 level are bolded.

**Table 3:** Pain and psychosocial outcomes by total body image percentage.

	TBI < Median (9.8%) (N = 191)	TBI > = Median (9.8%) (N = 168)	P-value
**Avg 0 –10 pain, daily diary**			
Mean (SD)	3.76 (2.54)	5.07 (2.32)	< 0.001
Median [Min, Max]	3.64 [0, 9.43]	5.20 [0, 9.50]	
Missing	33 (17.3%)	27 (16.1%)	
**Freq happy mood, daily diary**			
Mean (SD)	0.133 (0.199)	0.0885 (0.146)	0.0267
Median [Min, Max]	0 [0, 1.00]	0 [0, 0.889]	
Missing	33 (17.3%)	27 (16.1%)	
**PROMIS_Pain interference**			
Mean (SD)	60.5 (7.41)	65.0 (5.93)	< 0.001
Median [Min, Max]	61.5 [40.7, 77.0]	64.8 [40.7, 77.0]	
**ASCQ-Me Pain frequency**			
Mean (SD)	45.8 (13.2)	51.1 (10.7)	< 0.001
Median [Min, Max]	48.0 [20.8, 63.5]	51.8 [20.8, 63.5]	
**Pain severity**			
Mean (SD)	44.4 (14.5)	50.5 (11.0)	< 0.001
Median [Min, Max]	47.6 [14.9, 66.3]	52.3 [14.9, 66.3]	
**Pain catastrophizing**			
Mean (SD)	8.53 (4.19)	10.1 (3.89)	< 0.001
Median [Min, Max]	9.00 [0, 16.0]	11.0 [0, 16.0]	
**COMM Opioid Misuse**			
Mean (SD)	7.70 (4.98)	10.2 (5.60)	< 0.001
Median [Min, Max]	7.00 [0, 25.0]	10.0 [0, 29.0]	

Note: TBI = Total Body Image
